# Pharmacological inhibition of the MAP2K7 kinase in human disease

**DOI:** 10.3389/fonc.2024.1486756

**Published:** 2024-12-09

**Authors:** H. Daniel Lacorazza

**Affiliations:** Department of Pathology and Immunology, Baylor College of Medicine, Houston, TX, United States

**Keywords:** MAP2K7, kinase inhibitors, T-ALL, leukemia, mitogen-activated protein kinase kinase 7, cancer

## Abstract

The MAP2K7 signaling pathway activates the c-Jun NH2-terminal protein kinase (JNK) in response to stress signals, such as inflammatory cytokines, osmotic stress, or genomic damage. While there has been interest in inhibiting JNK due to its involvement in inflammatory processes and cancer, there is increasing focus on developing MAP2K7 inhibitors to enhance specificity when MAP2K7 activation is associated with disease progression. Despite some progress, further research is needed to fully comprehend the role of MAP2K7 in cancer and assess the potential use of kinase inhibitors in cancer therapy. This review examines the role of MAP2K7 in cancer and the development of small-molecule inhibitors.

## Introduction

Protein kinases are enzymes that catalyze the transfer of a phosphate group from ATP to specific amino acid residues (e.g., serine, threonine, and tyrosine) in target proteins. This process activates cellular pathways in response to various stimuli. The mitogen-activated protein kinases (MAPK) are Ser/Thr kinases that are part of eukaryotic signal transduction pathways that translate extracellular and intracellular stress signals to cellular responses by regulating gene expression in cell division, differentiation, and death. The conventional MAPK signaling units (e.g., ERK1/2, p38, JNK, and EKR5) are composed of a three-tier kinase cascade (MAP3K, MAP2K, and MAPK) stabilized by protein scaffolds (e.g., JNK interacting protein-1 or JIP1). Stress signals activate the upstream MAP kinase kinase kinase (MAP3K) that, in turn, sequentially phosphorylates and activates a downstream MAP kinase kinase (MAP2K) and downstream MAPK effectors (ERK1/2, p38, JNK, and EKR5) or MAPK-activated protein kinases (MAPKAPK). The activation strength depends on the duration and intensity of stimuli driven by extracellular factors sensed by cell surface receptors (e.g., growth factors, cytokines, and mitogens) and endogenous metabolic and DNA damage stress.

Abnormal activation of kinases has driven the development of small-molecule inhibitors for treating diseases. The catalytic domain contains a conserved core comprising two lobules (N-terminal and C-terminal) connected by a hinge region that defines the ATP-binding domain, which is the target of most kinase inhibitors. Kinase inhibitors can be classified into seven types (type I-VII) based on their binding (to the active ATP-binding site, inactive ATP-binding site, or different domain/allosteric) and binding mode (reversible or irreversible). The tyrosine kinase inhibitor Imatinib (classified as type IIA) was the inaugural small-molecule inhibitor to receive approval from the Food and Drug Administration (FDA) in 2001. Its successful management of patients with chronic myeloid leukemia marked a significant milestone in cancer therapy and paved the way for the development of various kinase inhibitors. The FDA has approved 72 such drugs, which collectively target approximately 12% of the kinome (comprising over 518 protein kinases) (1). MAP2K7 has emerged as a promising therapeutic target for pharmacological inhibition. The availability of crystal structure, chemical probes, and cell-free assays for MAP2K7, a member of the STE kinase family in the kinome, facilitates the development of specific inhibitors by medicinal chemistry. This review summarizes the current understanding of MAP2K7-driven JNK activation as a potential therapeutic target.

## The MAPK unit MAP2K7-JNK

The *MAP2K7* (a.k.a. MKK7, MEK7) gene (14 exons) encodes for a conserved regulatory dual specificity kinase of the JNK signaling cascade. Transcription of the *MAP2K7* gene can generate six isoforms through alternative splicing, named with the Greek alphabet (α1, α2, β1, β2, γ1, γ2) or variants 1-6 (2). MAP2K7α lacks an N-terminus fragment conserved in the other isoforms (3). Although the functional role of each isoform remains unclear, it was shown that T cell activation promotes a spliced isoform that restores the JNK-docking site by skipping the exon 2 (4). The full-length protein contains three conserved D-motifs in the N-terminus (docking of substrates), the kinase domain phosphorylated in the SXKAT motif by upstream kinases, and the DVD domain in the C-terminus (5).

**Figure 1:**
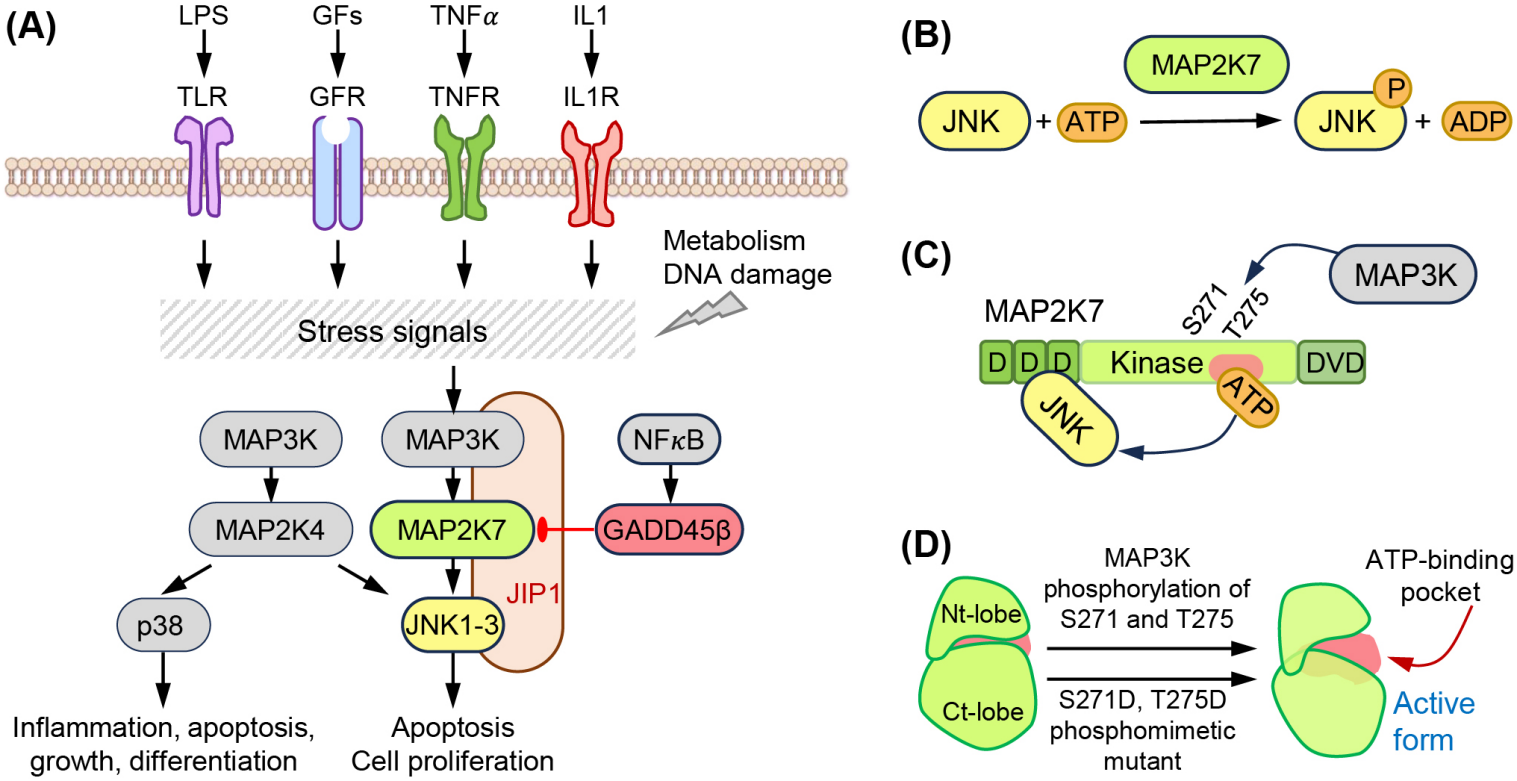
The MAP2K7-JNK pathway. **(A)** Extracellular (LPS, growth factors, TNFα, IL1) and intracellular (metabolism, DNA damage) stress signals activate the MAP3K-MAP2K7-JNK pathway. This is a three-tier kinase cascade stabilized by the scaffolding protein JIP-1. NF𝜅B activates the inhibitor GADD45β. **(B)** Structural domains of MAP2K7 proteins, D domains, kinase domain with Ser 271 (S271) and Thr 275 (T275) phosphorylated by MAP3K, and DVD domains. **(C)** Phosphorylation of S271 and T275 induces a conformation change, increasing accessibility to the ATP binding domain. **(D)** Phosphomimetic mutation activates MAP2K7. The phosphomimetic mutant S271D and T275D mimic the active form of MAP2K7.

MAP2K7 is part of the three-tiered signaling unit MAP3K (e.g., ASK1, TAK1), MAP2K (MAP2K7), and MAPK (JNK) that contributes to tissue homeostasis and responds to stress signals (**Figures 1A, B**) (5). Seven MAP2K proteins named MAP2K1 to MAP2K7 define the signaling units based on the downstream substrates (e.g., ERK1/2, JNK, p38, or ERK5). Specifically, MAP2K1 and MAP2K2 phosphorylate ERK1/2, MAP2K4 and MAP2K7 phosphorylate JNK, MAP2K3, MAP2K4, and MAP2K6 phosphorylate p38, and MAP2K5 phosphorylates ERK5. While MAP2K4 activates both JNK and p38, JNK is considered the sole substrate of MAP2K7, although it has been shown that MAP2K7 could activate p38 in macrophages (6). The upstream MAP3K, poorly defined, would bind to the MAP2K7 DVD domain and phosphorylates Ser 271 and Thr 275 in the SXAKT motif, causing a conformational change and increasing accessibility to the active site. This interaction leads to the phosphorylation of JNK, which is bound to the D domains in the N-terminus of MAP2K7 (**Figures 1C, D**). Although JNK does not phosphorylate a downstream MAPKAPK, JNK prevents DLK ubiquitination through phosphorylation in a potential feedback regulation (7). JNK phosphorylates transcription factors involved in gene regulation to respond to stress stimuli (e.g., ATF2, c-Jun). The MAP3K-MAP2K7-JNK complex is stabilized by scaffolding proteins, such as JNK interacting proteins (JIP1, JIP2, JIP3), modulating the intensity of the elicited signal.

Reports suggest that MAP2K7 activity is regulated via miRNAs and metabolism. Treatment of glucocorticoid-resistant CCRF-CEM cells with rapamycin, alone or combined with methylprednisolone, was associated with the upregulation of miR-331-3p and inhibition of the MAP2K7 pathway (8). Although the receptor tyrosine kinase inhibitor Sunitinib caused cardiotoxicity in old rats, the drug led to upregulation of miR-133b and inhibition of MAP2K7 in young rats (9). Amino acid deprivation induces ATF2 phosphorylation by activating GTPase Rac1/Cdc42 via Gα12 and MAP2K1/MAP2K7/JNK2 signaling to adapt to amino acid scarcity (10). The TOR signaling pathway regulator-like protein (TIPRL), upregulated in hepatocellular carcinoma cells, inhibits MAP2K7-JNK activation through binding of TIPRL to MAP2K7 and protein phosphatase type 2A (PP2Ac), causing dephosphorylation of MAP2K7 and preventing TRAIL-induced apoptosis (11).

Although JNK could be activated downstream of the T cell receptor (TCR), the function of MAP2K7 in T cells has yet to be thoroughly investigated. During thymic negative selection, immature CD4 and CD8 double-positive T cells activate JNK via MAP2K7 (12). The role of MAP2K7 in the JNK activation upon TCR crosslink in naïve CD4 T cells was disregarded due to undetectable levels (13). However, MAP2K7 is required for JNK activation and LPS-induced cytokine production in macrophages (6). In the immune response, the avian coronavirus induces a JNK-dependent pro-apoptotic activity through MAP2K7 upregulation in patients with post-COVID-19 infection (14, 15).

In addition to homeostatic and physiological regulation, the MAP2K7-JNK pathway can be modulated with small molecules. Treatment with luteolin, a natural product found in fruits and vegetables, attenuates the hepatic and adipocyte fibrosis in high fat-fed mice via the toll-like receptor (TLR) signaling, which was associated with increased expression of MAP2K7 among other MAPKs downstream of TLR5 signaling (16). The nucleoside analog cordycepin inhibits GADD45β by suppressing NF-𝜅B, resulting in the upregulation of MAP2K7 and JNK phosphorylation in renal cancer cells (17). Cobaltic protoporphyrin IX chloride (Rosiglitazone), an anti-diabetic medication, has a neuroprotective effect by inducing heme oxygenase that prevents the assembly of the MELK3-MAP2K7-JNK3 complex via JIP1 and thus heme oxygenase can be beneficial in cases of cerebral ischemia (18).

DNA damage by ionizing radiation activates the MAP2K7-JNK pathway through PKCδ (15). Oncogenic stress can also trigger DNA damage during replication, which induces p53-mediated apoptosis by activating the MAP2K7-JNK pathway (19). The complex formed by the glucocorticoid receptor and its ligand inhibits MAP2K7 by dissociating JNK and translocating it to the nucleus; however, this complex cannot promote transcription with JNK (20). The control of programmed cell death during homeostasis and carcinogenesis by NF-𝜅B involves activation of GADD45β, which inhibits MAP2K7 via protein-to-protein interaction that hinders access to the ATP binding site (21).

## Loss of function MAP2K7 mouse models reveal physiological functions

Loss-of-MAP2K7 models have provided information on its function in different tissues (**Table 1**). Although homozygous gene deletion causes embryonic lethality, the study of viable heterozygous mice allowed their use as a model of mono-allelic deletion of the *MAP2K7* gene in humans (23). The functional role of MAP2K7 in lymphoid and mast cells was evaluated in chimeric mice generated with recombination activating gene (Rag1) blastocyst complementation (**Table 1**) (22). Loss of MAP2K7 caused hyperproliferation of T and B lymphocytes in response to antigen receptor stimulation, which was associated with reduced expression of JunB and p16 and upregulation of Cyclin D1 (22).

**Table 1:** Mouse models of MAP2K7 genetic loss.

GEMM model	Gene deletion	Finding	Ref
*Map2k7* ^−/−^	Embryonic gene deletion	*▪Decreased proliferation (G2/M arrest) and premature senescence in mouse embryonic fibroblasts*	(68)
*Map2k7* ^+/−^	Embryonic gene deletion	*▪Cognitive impairment* *▪Attention deficit*	(69)
*Map2k7* ^neo/hyg^ and *Map2k7* ^+/neo^ somatic chimeras	Complementation chimeras	*▪Reduced JNK activation in mast cells* *▪Increased growth factor and antigen receptor-driven proliferation of hematopoietic cells*	(22)
*Map2k7* ^fl/fl^ Synapsin-Cre	Conditional deletion in neurons	*▪Impaired circadian rhythm, decreased locomotor activity, axonal degeneration in the spinal cord* *▪Depression like behavior*	(26) (27)
*Map2k7* ^fl/fl^ nestin-cre	Conditional deletion in the nervous system	*▪Perinatal lethality* *▪Reduced axon elongation and radial migration in the developing brain*	(28)
*Map2k4* ^fl/fl^ *Map2k7* ^fl/fl^ actin-CreERT2	Inducible deletion in adult mice	*▪Alterations in dendritic structure* *▪;Reduced JNK activation*	(70)
*Map2k4* ^fl/fl^ *Map2k7* ^fl/fl^ Six3-cre	Conditional deletion in the retina	*▪Alteration in retinal structure* *▪Disruption of inner nuclear layer cell somal and synaptic organization*	(29)
*Map2k7* ^fl/fl^ myosin light chain-Cre	Conditional deletion in cardiomyocyte	*▪Deterioration in ventricular function after pressure overload* *▪Increased cardiomyocyte apoptosis*	(30)

A wealth of studies indicates that MAP2K7 regulates functions in the central nervous system. JNK3 activity in cortical neurons led to research on the involvement of MAP2K4 and MAP2K7 in neurological disorders (23, 24). The impact of MAP2K7 haploinsufficiency on behavioral tasks was studied because post-mortem brain analysis showed lower MAP2K7 expression in individuals with schizophrenia compared to brain tissue from healthy individuals (**Table 1**) (4). The genetic association between MAP2K7 and schizophrenia correlated with impaired working memory in *Map2k7* heterozygous mice (25). The development of *Map2k7*-floxed mice allowed conditional gene deletion in different tissues through tissue-specific Cre-recombinase transgenic mice. Mice with conditional deletion of the *Map2k7* gene in neurons, using synapsin-cre mice, displayed macrocephaly, impairment of circadian rhythms, and progressive motor dysfunctions associated with axonal neuropathy and muscle atrophy (**Table 1**) (26). Another group reported that loss of MAP2K7, using the same conditional gene deletion approach, did not alter locomotor functions and cognitive capacity; however, the mice presented social depression-like behavior (27). Nestin-cre-driven *Map2k7* gene deletion in neural stem cells and postmitotic neurons resulted in lethality at birth (28). Post-mortem analysis of brains from *Map2k7* ^fl/fl^ nestin-cre mice showed large ventricles, reduced striatum, reduced axon formation positive for the transient axonal glycoprotein-1 in different areas, and defects in axon elongation and radial migration of neurons in the developing brain (28). Dual conditional deletion of the *Map2k4* and *Map2k7* genes using actin-Cre-ERT2 alters neuroblast migration and differentiation through a reduced JNK activation (28). Double deficiency in the retina caused a defect in retinal development and axonal injury-induced retinal ganglion cell death (29).

MAP2K7 has a protective function in cardiomyocytes. Conditional deletion of the Map2k7 gene in *Map2k7* ^fl/fl^ myosin-cre (**Table 1**) revealed that MAP2K7 promotes cardiomyocyte survival, suppressing extracellular matrix deposition and inhibiting hypertrophic growth, and thus preventing heart failure in response to pressure overload (30).

## Role of MAP2K7 in cancer

The impact of aberrant activation of the MAP2K7-JNK pathway in human disease has yet to be thoroughly investigated, even though JNK is implicated in diverse physiological processes and carcinogenesis. Since JNK is involved in cellular processes targeted in cancer and JNK is the sole substrate of MAP2K7, MAP2K7 emerges as a new potential therapeutic target. Studies on mouse models suggest that the MAPK pathway likely plays a role in cancer progression, metastasis, and resistance to chemotherapy.

The generation of mice carrying a *Map2k7*-floxed allele by Penninger’s group has led to studies on the role of MAP2K7 in solid tumors (**Table 2**) (31). For example, inactivation of MAP2K7 revealed a tumor suppressor function in two models of epithelial lung carcinomas (KRas^G12D^) and mammary tumors (NeuT) (31). The induction of lung cancer through inhalation of Cre-adenovirus in *Map2k7* ^fl/Δ^ *Lox-Stop-Lox-KRas* ^G12D^ mice shows accelerated cancer initiation and growth of lung adenomas with a rapid demise of tumor-bearing mice with 100% penetrance. Because MAP2K7-JNK stabilizes p53 through phosphorylation, the DNA damage response mechanism is activated in early lung lesions with low p53 levels. *Map2k7* deletion in mammary epithelial cells in *Map2k7* ^fl/Δ^ MMTV-Cre^+^ mice showed normal epithelial morphology but exhibited an early onset of mammary tumors associated with impaired p53 protein stability when crossed to MMTV-NeuT mice (31).

**Table 2:** Functional role of MAP2K7 in cancer models.

Disease	Role of MAP2K7	Pre-clinical model	Ref
Breast cancer	Tumor suppressor function	Acceleration of tumor onset in NeuT-driven mammary tumors *Map2k7* ^fl/fl^ K5-Cre.	(31)
Lung carcinoma	Tumor suppressor function	*Reduced survival to K-Ras induced lung carcinoma in the Map2k7* ^fl/fl^ lox-stop-lox-KRas^G12D^ and adenovirus-Cre model.	(31)
Liver metastasis of colon cancer	Pro-oncogenic	Mediates miR-493 suppression of liver metastasis of colon cancer cells.	(32)
Pancreatic ductal adenocarcinoma (PDAC)	Tumor suppressor function	Dual loss-of-function of MAP2K4 and MAP2K7 cooperates with Kras(G12D) to accelerate invasive PDAC. JNK inhibits Kras(G12D)-induced acinar to ductal metaplasia.	(34)
Glioblastoma	Pro-oncogenic	HDAC6 inhibition induces repression of MAP2K7 and JNK. HDAC4 deacetylates SP1 and KLF5 upregulating MAP2K7.	(35, 36)
Prostate cancer	Pro-oncogenic.	Increased levels of MAP2K4, MAP2K6, and MAP2K7 in mouse prostate TRAMP model and tissues from patients with high-grade prostatic intraepithelial neoplasia.	(38)
Lung squamous cell carcinoma (LSCC)	LKB1-mediated tumor suppression	Loss of LKB1 induces LSCC by reducing MAP2K7 levels and JNK1/2 activation.	(40)
MBNL1-low cancers	MBNL1 promotes exon 2 skipping in MAP2K7	MBNL1 and MAP2K7Δexon2 promote cancer stemness and increased susceptibility to JNK inhibition.	(41)
Leukemia	Pro-oncogenic.	KLF4 epigenetic silencing leads to de-repression and aberrant activation of MAP2K7-JNK in pediatric T-ALL.	(50)

Analysis of genes downregulated by miR-493 in the colon cancer cell line HCT116 allowed the prediction of potential targets through a combination of *in silico* programs. The miR-493 inhibits colon cancer metastasis in the lung by targeting the IGF1R and MAP2K7 transcripts (32). Although genomic silencing of MAP2K7 in HCT116 cells inhibited the foci formation in the liver, the mechanism of MAP2K7-driven liver metastasis has not been further investigated. Notably, the MAP2K7 p.Glu116Lys variant has been associated with lung cancer cell proliferation, tumor growth, and metastasis; however, the link between this variant and colon cancer metastasis remains unclear (33).

Pancreatic ductal adenocarcinoma (PDAC) is a devastating cancer with a poor prognosis. *Map2k4* ^fl/fl^ *Map2k7* ^fl/fl^ Pdx1-CreER mice carrying the Kras^G12D^ knock-in allele were generated to induce dual gene deletion by administering tamoxifen in the lactating dam and studying the role of JNK activity in PDAC pathogenesis (34). Dual deletion of Map2k4 and Map2k7 did not alter the tissue structure but led to accelerated development of abnormal cell growth driven by KrasG12D in the pancreatic lining. This resulted in widespread highly dysplastic ductal structures, intense stromal desmoplasia, and accelerated progression of PDAC with high penetrance and short latency (34). It is worth mentioning that individual deletion of MAP2K7 in the Kras^G12D^ model did not accelerate the progression of pancreatic cancer, suggesting that both kinases are required to suppress Kras^G12D^-induced reprogramming of acinar cells into duct-like cells. In a model of inflammatory ductal metaplasia, mice with the dual loss of MAP2K4 and MAP2K7 could not efficiently resolve pancreatitis, leading to only partial acinar regeneration (34). This is intriguing, considering the link between inflammation and carcinogenesis. This data supports the therapeutic relevance of pharmacological inhibition of MAP2K4 and MAP2K7 in pancreatitis and PDAC.

Pharmacological inhibition of histone deacetylase 6 (HDAC6) prevents the progression of glioblastoma multiforme, an aggressive form of cancer affecting the central nervous system with a poor prognosis. Inhibition of HDAC6 reduces glioma cell proliferation and invasion by destabilizing MAP2K7 protein, decreasing JNK and c-Jun activation, and subsequently downregulating cyclin D1 and matrix metalloproteinases (35). Furthermore, it was determined through genomic silencing that MAP2K7, not MAP2K4, phosphorylates JNK/c-Jun in glioma cells. The fact that HDAC4 and MAP2K7 are expressed at high levels suggests that the cancer-promoting effects of MAP2K7 in glioma cells could be stopped using drugs that inhibit HDAC4. In this scenario, inhibiting HDAC4 reduces the acetylation of the SP1 and KLF5 transcription factors, leading to decreased expression of MAP2K7 (36). These reports indicate that HDACs may regulate MAP2K7 expression in glioma cells by controlling transcription and protein stability.

The transcription factor c-Jun, a target of JNK activity, contributes to etoposide-induced apoptosis in prostate cancer cells by activating the death receptor FAS (37). Histopathological analysis revealed the presence of kinases MAP2K4, MAP2K6, and MAP2K7 in human prostate adenocarcinoma and neoplastic tissues from TRAMP mice but not in benign glands (38). While this finding does not establish a cause-effect relationship, the aberrant activation of these kinases provides an opportunity to investigate the use of specific small molecule inhibitors to inhibit prostate tumor growth. In addition, MAP2K7 also mediates signals from the discoidin domain receptor 1 (DDR1) and promotes the epithelial-mesenchymal transition during prostate cancer metastasis (39).

Finally, a study of an upstream regulator indirectly links MAP2K7 to cancer. Deleting the Lkb1 gene, which is often mutated in lung squamous cell carcinoma (LSCC), is sufficient to induce LSCC through the inactivation of the MAP2K7-JNK1/JNK2 pathway, rather than the AMPKα and mTOR pathways (**Table 2**) (40). This finding suggests that LKB1 regulates the MAP2K7-JNK pathway, at least during lung carcinogenesis. Another example is the master splicing regulator MBNL1. Low levels of MBNL1 were linked to poor survival in metastasis in triple-negative breast, lung, and gastric adenocarcinomas. Transcriptome analysis of isoforms in the stomach cell line HFE-145 with genomic silencing of MBNL1 identified a short list of alternative splicing events and genes regulated by transcript stability in MBNL1-regulated tumors. Loss of MBNL1 led to skipping exon 2 in the *MAP2K7* gene with generation of the MAP2K7Δexon2 splice variant responsible for increased stem/progenitor-like properties because of increased JNK signaling driven by a higher affinity of JNK to bind MAP2K7 (41). This study suggests that MBNL1 suppresses tumors by preventing the oncogenic MAP2K7 splice variant generation.

## Aberrant activation of MAP2K7 in pediatric T-ALL

As discussed above, most of the knowledge on the carcinogenic role of MAP2K7 relates to solid tumors (**Table 2**). Our group discovered that the MAP2K7-JNK pathway is aberrantly activated in children with T-cell acute lymphoblastic leukemia (T-ALL). ALL is the most common cancer in children under 14 years of age, and it is classified depending on the lymphocytes involved, such as B-ALL (B cells) and T-ALL (T cells) (42–45). Treatment modalities have significantly improved outcomes in specialized centers, with 5-year relapse-free survival rates greater than 80-85% (46). Unfortunately, the prognosis is not as favorable for children whose treatment fails to induce long-lasting remission, with the event-free survival rate dropping to around 30% in subsequent treatments (47). As a result, leukemia continues to be a significant cause of cancer-related deaths in children, especially for those with refractory or relapsed disease. This highlights the critical need for alternative drugs to improve survival rates in frontline and salvage therapies for leukemia (47–49).

**Figure 2:**
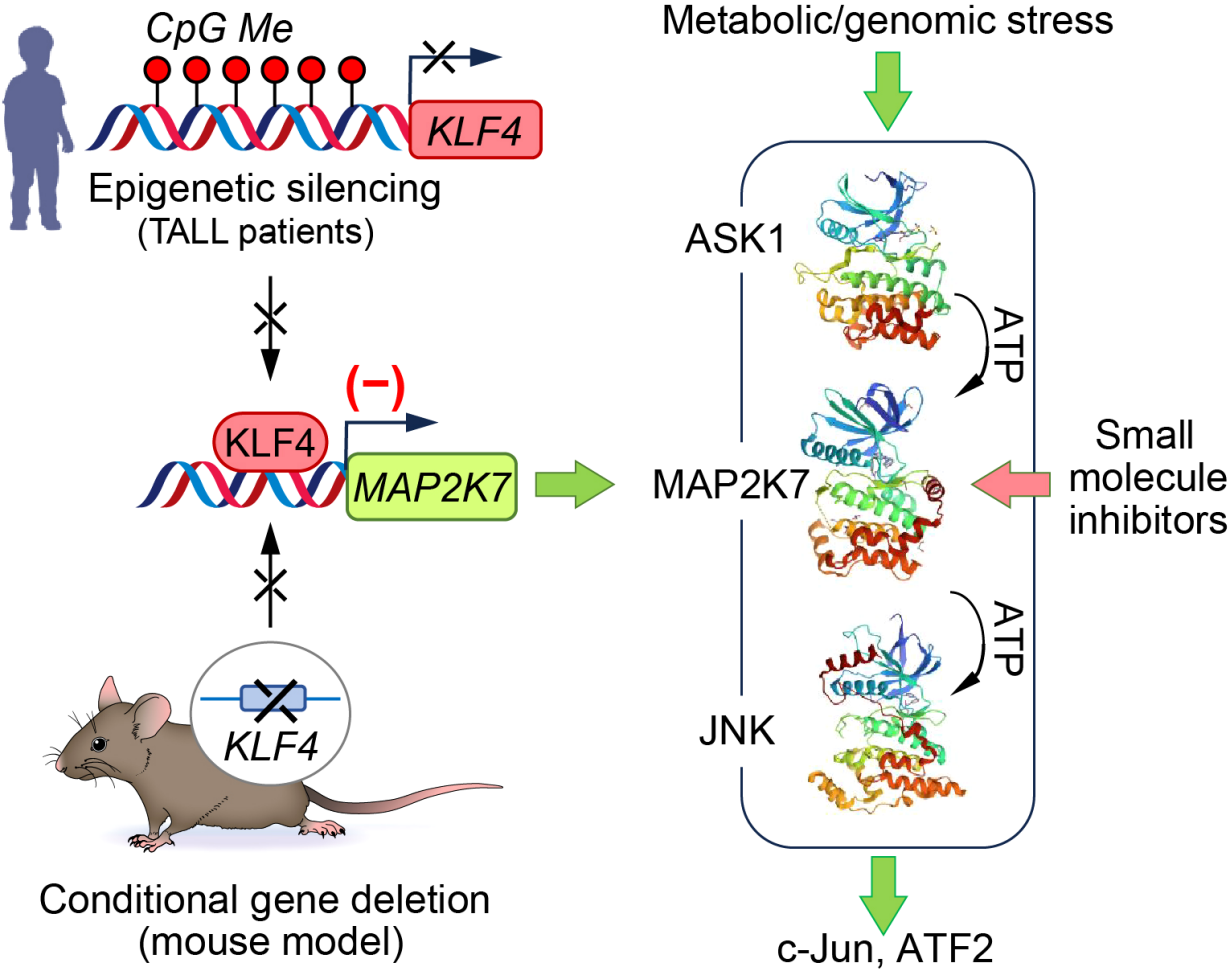
Aberrant MAP2K7 activation in pediatric T-ALL. Samples from pediatric T-ALL patients showed epigenetic silencing of KLF4 via CpG methylation with elevated activation of the MAP2K7-JNK pathway. The conditional deletion of the *Klf4* gene in the NOTCH1-induced T-ALL model recapitulates the findings in human samples showing upregulation of MAP2K7 and activation of the MAP2K7-JNK pathway that drives the proliferation of leukemia-initiating cells and leukemic T-ALL cells.

We studied the tumor suppressor function of the Krüppel-like factor 4 (KLF4) in pediatric leukemia because we identified that KLF4 inhibits T cell proliferation during homeostasis, and it is expressed at low levels in lymphoblasts from children with T-ALL, especially in the poor prognoses of ETP-ALL and TLX groups and the inhibitory role in T cell proliferation (50–52). Consistent with these findings, NOTCH1-induced T-ALL mice with conditional deletion of the *Klf4* gene displayed more aggressive leukemia, associated with increased expansion of leukemia-initiating cells (LICs) determined by immunophenotypic analysis and limiting-dose transplantation. Mechanistically, the acceleration of leukemia was linked to the upregulation of the mitogen-activated kinase MAP2K7 because the loss of KLF4 released repression of the *Map2k7* gene (**Figure 2**). Consistent with these findings in the mouse model, analysis of lymphoblasts from children with T-ALL showed epigenetic silencing of the *KLF4* gene by CpG methylation, low KLF4 expression, and elevated levels of total and phosphorylated MAP2K7 protein (**Figure 2**). The fact that the activated MAP2K7-JNK pathway augmented the proliferation of bulk leukemic cells and LICs suggests that MAP2K7 inhibition represents a novel approach to eradicating LICs thought to drive chemoresistance and relapses. Analysis of the ShinyDepMap database shows a low MAP2K7 dependency (−0.575) because it contains mainly AML and B-lymphoma cell lines (53). According to the cell line Encyclopedia (CCLE), MAP2K7 is highly expressed in multiple myeloma, chronic myeloid leukemia, lymphomas, B-cell acute lymphoblastic leukemia (B-ALL), and T-cell acute lymphoblastic leukemia (T-ALL). Further, proteomic analysis revealed higher levels of MAP2K7 expression in acute lymphoblastic leukemia (ALL), lymphoma, and myeloma compared to other cancer types.

## Pharmacological modulation of MAP2K7-JNK activity

There is an increasing interest in developing MAP2K7 inhibitors not only to treat human disease but also as a tool to investigate MAP2K7 in T-ALL pathobiology. Specific inhibitors are needed because most small molecules used to study MAP2K7, with a few exceptions, were identified as off-target inhibitors. Although medicinal chemistry aims to increase specificity through covalent inhibition, clinical oncologists are concerned that covalent inhibitors may cause off-target toxicity. Despite this, many kinase inhibitors used in the clinic covalently bind to the target. The arduous path of drug discovery is fraught with challenges, including high costs, low clinical efficacy, toxicities, and poor pharmacokinetics that decrease the likelihood of clinical success. However, the potential benefits outweigh the inherent risks.

Based on the interaction of the NF𝜅B-regulated antiapoptotic factor GADD45β and MAP2K7, screening a combinatorial library of L-tetrapeptides to identify peptides capable of disrupting the MAP2K7:GADD45β complex resulted in the development of the D-tripeptide (DTP3). Selected compounds with low IC50 were further optimized for stabilization (D-enantiomers), cell membrane permeability (replacing the N-terminal acetyl group with a benzyloxycarbonyl group), and chemical derivatization to improve bioavailability while retaining anticancer activity. DTP3 retained the capacity to induce apoptosis with high potency by allosterically disrupting the MAP2K7:GADD45β interaction. The survival of multiple myeloma (MM) malignant plasma cells is driven through MAP2K7-JNK inhibition by GADD45β activated by constitutively activated NF-𝜅B. Hence, DTP3 induces apoptosis by releasing inhibition of MAP2K7 by GADD45β and reactivating the MAP2K7-JNK pathway in MM cells (54). The effect of DTP3 on MAP2K7 activity is the only known example where MAP2K7-JNK activation instead of inhibition has antileukemic properties (**Table 3**).

**Table 3:** Pharmacological modulation of MAP2K7 activity.

Chemical compound	Specificity	MAP2K7 inhibition (IC50)	Cytotoxicity IC50	Cancer	*A*ctivity in a mouse model (dose)	Ref.
DTP3 peptide	Inhibits interaction of GADD45β and MAP2K7	Activation (28 nM)	17-31 nM	MM	+++ (14.5 mg/Kg)	(54)
HWY336	MAP2K4, MAP2K7	Inhibition (10 μM)	n.d.	n.d.	n.d.	(55)
Cov-2 Cov-4 Cov-3	MAP2K7 (covalent)	Inhibition (11 nM, 502 nM, 873 nM)	n.d.	n.d.	n.d.	(71)
4-aminopyrazolo pyrimidine-based inhibitors	MAP2K7, EGFR	Inhibition (10 nM)	n.d.	n.d.	n.d.	(56)
Ibrutinib	BTK, MAP2K7	Inhibition (160 nM)	>5 μM	CLL	n.d.	(57)
5Z-7-Oxozeaenol	MAP2K7 (covalent)	Inhibition (1.2 μM)	0.2-1.1 μM	T-ALL	+ (15 mg/Kg)	(64)
OTSSP167	MELK, MAP2K7 (pan-kinase inhibitor)	Inhibition (160 nM)	10-57 nM	T-ALL	+++ (10 mg/Kg)	(65)
Compound 1 and Compound 2	MAP2K7 (covalent)	Inhibition [3 nM (1), 0.6 nM (2)]	n.d.	OS	n.d.	(66)
DK-2403	MAP2K7 (covalent)	Inhibition (10 nM)	1.1-2.9 μM	T-ALL	n.d.	(67)

nd, not determined.

MM, multiple myeloma; CLL, chronic lymphocytic leukemia; T-ALL, T-cell acute lymphoblastic leukemia; OS, osteosarcoma.

Next, we will summarize the development of MAP2K7 inhibitors for therapeutic use. HWY336 was identified in a protoberberine compounds chemical library screen as a MAP2K4 and MAP2K7 kinase inhibitor in the human embryonic kidney HEK293 cell line (**Table 3**) (55). HWY336 competes with substrates but not with ATP via non-covalent interactions in the activation loop of MAP2K4 and MAP2K7, yet this inhibitor’s anticancer properties have to be investigated. A structure-based design was conducted using the structure of an EGFR inhibitor able to inhibit MAP2K7 with low potency because pyrazolopyrimidine-based compounds inhibit EGFR, and the ATP binding pocket of EGFR is similar to MAP2K7 (**Table 3**) (56). The compound 4a showed the highest potency (10 nM) in a biochemical assay and inhibited MAP2K7 by covalently reacting to Cys218 at the end of the hinge region in the ATP binding domain. Kinome studies of compound 4a revealed off-target kinases with more than 50% inhibition (e.g., BLK, BMX, BTK, ITK, JAK3, mTOR, and S6K) all containing the Cys218 in the ATP-binding pocket (56). It is essential to highlight that only 11 kinases in all the kinome have cysteine in position 218. Compound 4a showed suitable pharmacokinetics parameters that are encouraging for *in vivo* studies.

A recent study showed a structural analysis of MAP2K7 activation and identified small molecules with inhibitory activity (57). The wild-type ΔN115-MAP2K7 protein in the non-phosphorylated form and the phosphorylation-mimetic mutant, substituting Ser287 and Thr291 for aspartic acid, adopted an inactive state with a closed ATP binding site (57). An active conformation was achieved by eliminating the N-terminal helix in ΔN75-MAP2K7-S287D/T291D crystals, suggesting that the N-terminal regulates the kinase activation (57). Then, a small-scale screen (360 compounds) using the melting thermal shift assay identified nine compounds that could bind the flexible MAP2K7 ATP binding pocket with K_D_ in the nanomolar range. These compounds included type I inhibitors (e.g., Ibrutinib, OTSSP167, and CPT1-70-1) and trifluoromethyl-benzene-based type II inhibitors (e.g., HYJ2-002-1, XMD15-46, and TL10-105) (57). It has been postulated that Ibrutinib inhibits MAP2K7 by forming a covalent adduct with Cys 218 and through allosteric binding. Kinase assays using full-length MAP2K7-S287D/T291D protein with ATP pre-incubation showed high potency for the nine identified compounds with IC50 values ranging from 60 nM to 160 nM. The compounds that showed higher cytotoxicity in the monocytic cell line THP-1 at 1 μM concentration were OTSSP167, CPT1-70-1, and TL10-105. In this assay, THP-1 cells were pre-treated with sorbitol to activate MAP2K7-JNK because this pathway is not activated in THP1 cells, as we have shown for T-ALL cell lines (50). This report has shed important information on the MAP2K7 structure in the inactive and active form, the flexibility of the ATP binding domain, and the inhibitory activity of Type I and Type II reversible and covalent inhibitors.

Our group decided to investigate whether the MAP2K7-JNK pathway could be a druggable target to treat pediatric T-ALL. JNK inhibitors (e.g., JNK-IN-8) showed cytotoxicity in T-ALL cell lines with IC50 > 10 μM by inhibiting the MAP2K7-JNK pathway (58, 59). Even if JNK inhibition reduced the expansion of human leukemia cells in a cell-based xenograft model, low specificity and potency prevented reaching sustained therapeutic concentrations with minimal toxicity (50, 60). We hypothesize that direct MAP2K7 inhibition could increase specificity to T-ALL, considering that MAP2K7 and MAP2K4 can activate JNK. Selective inhibition of the kinase MAP2K7 with small molecules is possible because of the distinct features of its ATP binding pocket compared to other MAP2Ks (61, 62). For instance, four cysteine residues, including Cys 218, exist in the hinge region. The fungal natural product 5Z-7-Oxozeaenol, which covalently reacts with the Cys 218 (62, 63), induced cytotoxicity in T-ALL cell lines with IC50s ranging from 0.2-1.1 μM by inducing G2/M cell cycle arrest and apoptosis (64). 5Z-7-Oxozeaenol synergistically induces *in vitro* cytotoxicity with etoposide and dexamethasone, suggesting the feasibility of using this inhibitor in frontline therapy. However, this compound failed to efficiently control leukemia in the cell-based xenograft (CBX) and patient-derived xenograft (PDX) models due to drug toxicity that prevented achieving therapeutic concentrations *in vivo* (64). We recently found that the MELK inhibitor OTSSP167 also inhibited MAP2K7 kinase activity at an IC50 of 160 nM and induced cytotoxicity in T-ALL cells at low nanomolar concentrations (IC50 10-57 nM) through inhibition of the MAP2K7-JNK pathway in T-ALL cell lines (57, 65). OTSSP167 inhibition was also evaluated upon MAP2K7 activation through genetic (ectopic expression of the MAP2K7-JNK fusion) or metabolic (sorbitol) approaches. Despite being a pan-kinase inhibitor, OTSSP167 targeted other pathways besides MAP2K7-JNK, such as mTOR and NOTCH1, in T-ALL cells (65). These off-target inhibitions could be beneficial because mTOR and NOTCH1 are critical in the T-ALL pathogenesis. Daily administration of OTSSP167 (10 mg/Kg) in T-ALL PDX mice, generated with remission and relapse clinical samples, showed efficient control of leukemia burden (65). Finally, OTSSP167 showed synergism when combined with drugs used in standard therapy (e.g., vincristine, asparaginase, dexamethasone), which is highly relevant for clinical translation because any new drug will be added as an adjuvant of the standard treatment. Yet, despite being potent and well tolerated, future use of OTSSP167 in the clinic is somehow overshadowed by the potential of target toxicity due to its pan-kinase inhibitor activity.

Recently, covalent MAP2K7 inhibitors were identified in a high-throughput nanomole-scale synthesis for late-stage functionalization of acrylamide-based kinase inhibitors (**Table 3**) (66). Two MAP2K7 inhibitors (compounds 1 and 2), previously identified via structure-based drug discovery and virtual screening, were used to generate libraries through copper-catalyzed azide-alkyne cycloaddition synthesis that were screened with the in-cell western assay (phosphorylated JNK detection) using U2O2 cells (osteosarcoma) treated with sorbitol. The generated series from compounds 1 and 2 yielded derivatives (4-amino-pyrazolpyrimidine core and indazole scaffold) with JNK inhibition capacity in the low nanomolar range (<30 nM) and irreversible binding to the Cys 218 in the recombinant protein. Although the top inhibitors in each series were selected for absorption, distribution, metabolism, and excretion studies, none of these compounds were tested in mouse models.

Finally, Scheidt’s group designed a novel MAP2K7 inhibitor, DK-2403, based on rational design with a streamlined one-pot synthesis (67). Besides MAP2K7, a screen of 90 kinases revealed that DK-2403 could bind EGFR at 1 μM, suggesting high target selectivity. This off-target activity is irrelevant for T-ALL because EGFR inhibition does not cause significant cytotoxicity. The specificity of DK-2403 to inhibit MAP2K7 is partly driven by the capacity of DK-2403 to bind to Cys 218 covalently, which was determined by liquid chromatography and time-of-flight mass spectrometry (LC-TOF MS) and confirmed by wash-out experiments. Cell viability assays showed IC50 cytotoxicity in the 1.1-2.9 μM range and reduced levels of phosphorylated JNK and ATF2 in the T-ALL cell lines JURKAT, KOPT-K1, RPMI-8402, ALL-SIL (67). DK-2403 has not yet been tested in leukemic mouse models.

## Discussion

The abnormal kinase activation in diseases makes it suitable to develop pharmacological inhibition through medicinal chemistry campaigns as a therapeutic approach. In recent years, significant efforts have been dedicated to developing novel MAP2K7 inhibitors for use as research tools and potential new cancer treatments. Among the nine small molecules with MAP2K7 inhibitory capacity described in the literature, only four were evaluated in cancer models, predominantly multiple melanoma and T-cell acute lymphoblastic leukemia, and three were assessed in mouse models. Our research group identified abnormal MAP2K7 activation in pediatric T-ALL and conducted proof-of-concept evaluations of 5Z-7Oxozeaenol and OTSSP167 compounds. Although promising results indicated that MAP2K7 inhibition can control leukemia burden in preclinical mouse models, the compounds did not exhibit the desired high potency, high specificity, and low toxicity. More recently, we described the design and synthesis of an irreversible MAP2K7 inhibitor, DK2403, with high specificity that has yet to be investigated in pre-clinical mouse models.

The clinical application of potent and specific MAP2K7 inhibitors in leukemia also requires optimal pharmacology and toxicology studies in animal models and pre-clinical evaluation using patient samples. The targeting of leukemia-initiating cells, which are responsible for refractory and relapsed disease, should also be evaluated in pre-clinical models of T-ALL. Because haploinsufficiency and conditional MAP2K7 gene deletion were associated with lymphocyte hyperproliferation, schizophrenia, neuropathy, and depression, systemic MAP2K7 inhibition may cause side effects due to off-tissue activity. Undesired activity in the central nervous system could be minimized by decreasing the lipophilicity of lead compounds or devising delivery systems that are not permeable in the blood-brain barrier. MAP2K7 inhibition should not alter lymphocyte function unless the patient has an underlying immune response, which could represent exclusion criteria in clinical trials. For all these reasons, Phase I clinical trials should examine the safety and adverse events of MAP2K7 inhibitors, particularly in cognition, cardiac function, and immunity. Drug combination studies are needed because MAP2K7 inhibition would be part of a multi-drug frontline or salvage treatment. In addition, additional research on the role of MAP2K7 in normal tissue homeostasis is necessary to predict the potential side effects and safety of pharmacological inhibition. Another alternative to overcome these shortcomings is repurposing existing kinase inhibitors tested in humans for new therapeutic purposes, although this would imply off-target inhibition of MAP2K7 (low potency). In addition to leukemia, MAP2K7 inhibition may also benefit cancers associated with increased activation of the MAP2K7-JNK pathway, such as liver metastasis of colon cancer, glioblastoma, and prostate cancer.

A clearer understanding of MAP2K7's role in normal and disease states and the development of small-molecule inhibitors will facilitate the exploration of potential clinical applications in cancer and other diseases.
